# Association between *IL28B* Polymorphisms and Spontaneous Clearance of Hepatitis B Virus Infection

**DOI:** 10.1371/journal.pone.0069166

**Published:** 2013-07-17

**Authors:** Seung Up Kim, Ki Jun Song, Hye Young Chang, Eui-Cheol Shin, Jun Yong Park, Do Young Kim, Kwang-Hyub Han, Chae Yoon Chon, Sang Hoon Ahn

**Affiliations:** 1 Department of Internal Medicine, Yonsei University College of Medicine, Seoul, Korea; 2 Institute of Gastroenterology, Yonsei University College of Medicine, Seoul, Korea; 3 Department of Biostatistics, Yonsei University College of Medicine, Seoul, Korea; 4 Liver Cirrhosis Clinical Research Center, Seoul, Korea; 5 Brain Korea 21 Project of Medical Science, Seoul, Korea; 6 Laboratory of Immunology and Infectious Disease, Graduate School of Medical Science and Engineering, KAIST, Daejeon, Korea; The University of Hong Kong, Hong Kong

## Abstract

**Background/Aims:**

Single-nucleotide polymorphisms (SNPs) near the interleukin 28B gene (*IL28B*; interferon [IFN]-λ-3) are associated with outcomes of chronic hepatitis C virus (HCV) and hepatitis B virus (HBV) infection treated with peginterferon (PEG-IFN) alpha-based antiviral therapy. In this study, we investigated the influence of *IL28B* polymorphisms on spontaneous clearance of HBV infection in a large Korean cohort.

**Methods:**

Between January 2007 and June 2010, a total of 208 patients with chronic HBV infection and newly diagnosed HBV-related hepatocellular carcinoma were recruited as the CC group [HBsAg(+) for >6 months, anti-HBc(+), and anti-HBs(-)]. In addition, 351 organ donors were stratified into the UE group [n = 106; HBsAg(-), anti-HBc(-), and anti-HBs(-)] or the SC group [n = 245; HBsAg(-), anti-HBc(+), and anti-HBs(+)]. The SNaPshot ddNTP Primer Extension Kit (Applied Biosystems, Foster City, CA) was used for SNP detection. Direct full sequencing of the *IL28B* coding region was attempted.

**Results:**

Regardless of group, *rs12979860* CC was most frequently identified (85.0% in UE, 85.9% in SC, and 93.5% in CC, respectively), whereas *rs12979860* TT was not identified in any group. Similarly, *rs12980275* AA and *rs8099917* TT were most frequently identified (≥85%) regardless of group, whereas *rs12980275* GG was identified in only one subject in the SC group. In addition, *rs8099917* GG was not identified. The prevalences of CC in *rs*12979860, AA in *rs*12980275, and TT in *rs*8099917 were significantly higher in the CC group when compared with the UE and SC group (all *P*<0.05). Among 19 novel SNPs in the *IL28B* coding region, the proportions of 6 SNPs were significantly different among the UE, SC, and CC groups (all *P*<0.05).

**Conclusions:**

The SNP upstream of *IL28B* that has the strongest genetic association with HCV recovery has an inverse influence on HBV recovery. Additional studies are needed to understand the mechanisms of this SNP in HBV infection.

## Introduction

Recently, several independent genome-wide association studies have shown that genetic polymorphisms at or near the interleukin 28B gene (*IL28B*; also known as interferon [IFN]-λ-3), including *rs12979860*, *rs12980275*, and *rs8099917*, are associated with higher rates of sustained virologic response (SVR) in patients with chronic hepatitis C (CHC) treated with peginterferon (PEG-IFN) alpha and ribavirin [1]–[4]. Although the mechanism by which *IL28B* influences the response to PEG-IFN therapy has remained elusive, it is likely that the relationship is not specific to hepatitis C virus (HCV) infection. Indeed, the type of IFN coded for by *IL28B,* IFN-λ, has been previously shown to be active against several other viruses including hepatitis B virus (HBV) [5], [6]. Recently, a human study also demonstrated that *IL28B* polymorphisms were independently related to serological responses to PEG-IFN in a large global cohort of patients with hepatitis B e antigen positive chronic hepatitis B (CHB), similar to the results from chronic HCV infection [7], [8].

In addition to this association between *IL28B* polymorphisms and treatment responses to PEG-IFN, the relationship between *IL28B* polymorphism and spontaneous clearance of HCV infection has also been reported in several previous studies [1], [4], [9]–[11]. However, only one study, as a brief report, investigated the association between *IL28B* polymorphism and spontaneous clearance of HBV infection. The study concluded that the single-nucleotide polymorphism (SNP) 4 kb upstream of *IL28B* (*rs12979860*), which has a strong genetic association with HCV recovery, had no association with spontaneous recovery from HBV infection [13]. Although it is unknown how *rs1297860* affects the activity of *IL28B*, the authors proposed that it presumably alters the immune function to HCV, but not HBV [12].

Therefore, the *IL28B* polymorphism seems to have similar influences on both the response to antiviral treatment and spontaneous clearance in HCV infection, whereas it exhibits different influences in HBV infection between treatment outcomes using PEG-IFN and spontaneous clearance [7], [12]. However, data on this issue are extremely rare in terms of explaining the different influences of *IL28B* polymorphism on treatment outcomes using PEG-IFN and spontaneous clearance in HBV infection [7], [12]. Furthermore, previous studies have only attempted to characterize the *IL28B* polymorphism upstream of the *IL28B* coding region. Thus, we first investigated the influence of the *IL28B* polymorphism on HBV infection in a large Korean cohort and evaluated whether there is any novel polymorphism in the *IL28B* genome, especially in the coding region.

## Materials and Methods

### Patients

Between January 2007 and June 2010, a total of 208 patients with CHB infection and newly diagnosed CHB-related hepatocellular carcinoma (HCC) were prospectively recruited at the Severance Hospital, Yonsei University College of Medicine, Seoul, Korea and defined as the CC group [HBsAg(+) for >6 months, anti-HBc(+), and anti-HBs(-)]. During the study period, a total of 351 subjects who were admitted as living liver or kidney donors were also prospectively recruited and stratified into the UE group (n = 106) or SC group (n = 245) according to their HBV serological markers. Subjects were stratified into the UE group when their HBV serological markers were HBsAg(-), anti-HBc(-), and anti-HBs(-), whereas subjects who were HBsAg(-), anti-HBc(+), and anti-HBs(+) were stratified into the SC group. All subjects were native Korean and provided written informed consent for participation and for use of their genetic material for this study. The study protocol conformed to the ethical guidelines of the 1975 Helsinki Declaration and was approved by the Institutional Review Board of Severance Hospital.

The exclusion criteria were as follows: (1) no available HBV serological markers, (2) vaccinated subjects who were positive for anti-HBs alone, but negative for anti-HBc, (3) HCV or human immunodeficiency virus coinfection, (4) presence of other chronic liver disease, such as autoimmune hepatitis, toxic hepatitis, or primary biliary cirrhosis, (5) immunosuppressants, chemotherapy, or systemic corticosteroids within 3 months at enrollment, and (6) not a native Korean.

### Laboratory Tests

All serum specimens were stored at −70°C before testing. Human immunodeficiency virus antibody was determined by enzyme-linked immunoassay (ELISA) with Western blot confirmation. HCV antibody and HCV RNA were assayed using commercially available kits. HBV serological markers for HBsAg, anti-HBc, and anti-HBs were conducted with ELISAs (Dade Behring, Marburg, Germany).

### *IL28B* Genotyping

Genomic DNA was extracted using a Qiagen DNA Blood Mini Kit (Qiagen, Mildren, Germany) according to the manufacturer’s instructions. Three primer sets were used to amplify three SNPs (*rs12979860*, *rs12980275*, and *rs8099917*) in the *IL28B* gene (**Table 1**). Reactions were screened by gel electrophoresis with ethidium bromide staining and the polymerase chain reaction (PCR) product was purified by a PCR purification kit (Qiagen, Mildren, Germany).

**Table 1:** Primer sequence and polymerase chain reaction condition.

Target name	Method	Primer sequence		Tm (°C)
*rs12979860*	SNaP shot	Forward	AGACAACCAGGGTGAAGCAA	65
		Reverse	CTGCTCGCAGCCTCAGTC	
		Genotyping	CGGAGYGCAATTCAACCCTGGTTC	
*rs12980275*	SNaP shot	Forward	GAGGAGGGAAGGAAGTTCT	60
		Reverse	AGGTCTGGTCCTAGTGGTG	
		Genotyping	CCCCGGCAAATATTTAGACACGTC	
*rs8099917*	SNaP shot	Forward	TCCATGTGTTTATTTGTGC	55
		Reverse	GGAGAATGCAAATGAGAGA	
		Genotyping	TACAGCATGGTTCCAATTTGGGTGA	
Promoter-1	Direct sequencing	Forward	GGTGGCCTGAGTTTCAGTTC	60
		Reverse	TGCCCAGAGGCCAATATTTC	
Promoter-2	Direct sequencing	Forward	CCTTCGTCACACCTCAATTC	61
		Reverse	GGAAGGTATGTTCCCAAGAG	
Promoter-3	Direct sequencing	Forward	GAGCAGGTGGAATCCTCTTG	60
		Reverse	CCCGGTCATGTCTGTGTC	
Exon-intron 1	Direct sequencing	Forward	GTGGGCAGCCTCTGCATTC	58
		Reverse	AGCAGAAGCGACTCTTCC	
Exon-intron 2	Direct sequencing	Forward	GGCTAACCTGTGCCTTTG	60
		Reverse	GGAGCTGGGAGAGGATATG	
Exon-intron 3	Direct sequencing	Forward	CTGACGCTGAAGGTTCTG	62
		Reverse	CAAATACATAAATAGCGACTGGGTGAC	
3′ UTR-1	Direct sequencing	Forward	CTTCCGCCAGTCATGCAAC	60
		Reverse	TCAAGTGATCCTCCCAACTC	
3′ UTR-2	Direct sequencing	Forward	CCTGGATGTGATTGCTCAAG	60
		Reverse	GGTGGAGAATGACACTCTG	
3′ UTR-3	Direct sequencing	Forward	TGAGCTGCTGGAACAAAG	62
		Reverse	AGCAGGCACCTTGAAATGTC	

UTR, untranslated region.

For detection of the three SNPs (*rs12979860*, *rs12980275*, and *rs8099917*) near the *IL28B* gene on chromosome 19, which was identified in previous studies, primer extension reactions were performed using SNaPshot ddNTP Primer Extension Kit (Applied Biosystem, Foster City, CA). These SNPs were chosen because they were previously reported in three independent studies including mostly white and Asian patients with chronic HCV infection [2], [3], [11]. To clean up the primer extension reaction, one unit of shrimp alkaline phosphatase was added to the reaction mixture, and the mixture was incubated at 37°C for 1 hour followed by 15 min at 72°C for enzyme inactivation. The DNA sample, containing extension products and the Genescan 120 Liz size standard solution, was added to Hi-Di formamide (Applied Biosystems, Foster City, CA) according to the manufacturer’s recommendations. The mixture was incubated at 95°C for 5 min followed by 5 min on ice. The results were analyzed using the ABI Prism GeneScan and Genotyper program (Applied Biosystems, Foster City, CA).

### Full Sequencing of the *IL28B* Coding Region

Sequencing of the *IL28B* coding region was performed using the primers listed in **Table 1**. Nine primer sets were used to amplify the *IL28B* coding region. PCR products were identified by electrophoresis, and PCR products were then purified with a PCR purification kit and direct sequencing by an ABI 310 automated sequencer (Applied Biosystems, Foster City, CA).

### Statistical Analysis

Data are expressed as the median (range), n (%), or n, as appropriate. Observed numbers of each genotype were compared with the expected values in order to test whether the sample was in Hardy-Weinberg equilibrium using the chi-Square test with one degree of freedom. Haplotypic association analysis was performed using PLINK version 1.06 (http://pngu.mgh.harvard.edu/purcell/plink/). The odds ratio (OR) was calculated to indicate the associated risk and presented with 95% confidence intervals (CI). A *P*-value <0.05 on a two-tailed test was considered statistically significant. Statistical analyses were performed with SPSS version 11.0 (SPSS, Inc., Chicago, IL).

## Results

### Baseline Non-genetic Data

The mean age of the subjects in the UE (53 men and 53 women), SC (135 men and 110 women), and CC groups (153 men and 55 women) were 38 (median, 38; range, 18–59), 42 (median, 43; range, 18–69), and 44 (median, 45; range, 18–68) years, respectively. Subjects in the UE group were significantly younger than those in the SC (*P* = 0.004) and CC groups (*P*<0.001). The proportion of male subjects in UE group (50.0%) was significantly lower than that in the SC (55.5%, *P*<0.001) and CC groups (73.6%, *P*<0.001). Genotypic analysis of HBV showed that all subjects in this study had genotype C. *IL28B* SNP had no association to age (*P*>0.05) and was not prominent in different age group (*P*>0.05 by one way ANOVA).

### Prevalence of *rs12979860*, *rs12980275*, and *rs8099917* in the UE, SC, and CC Groups

Excluding cases with failed genotypic analyses (0∼26.0%), genotypes were successfully called for *rs12979860* in 100 of 106 (94.3%) subjects in the UE group, 220 of 245 (89.8%) in the SC group, and 154 of 208 (74.0%) in the CC group, respectively; for *rs12980275* in 106 of 106 (100%) subjects in the UE group, 243 of 245 (99.2%) in the SC group, and 203 of 208 (97.6%) in the CC group, respectively; and for *rs8099917* in 106 of 106 (100%) subjects in the UE group, in 241 of 245 (98.4%) in the SC group, and in 204 of 208 (98.1%) in the CC group, respectively (**Table 2**).

**Table 2:** Prevalence of three SNPs (*rs12979860*, *rs12980275*, and *rs8099917*) in UE, SC, and CC groups.

SNP	UE (n = 106)	SC (n = 245)	CC (n = 208)	*P* value
				UE *vs*. CC/SC *vs.* CC
*rs12979860*				
Analyzed samples	100	220	154	
CC	85 (85.0)	189 (85.9)	144 (93.5)	0.039/0.013
CT	15 (15.0)	31 (14.1)	10 (6.5)	
TT	0 (0)	0 (0)	0 (0)	
C allele	185	409	298	0.030/0.025
T allele	15	31	10	
*rs12980275*				
Analyzed samples	106	243	203	
AA	90 (85.0)	208 (85.6)	185 (91.1)	0.018/0.042
AG	16 (15.0)	34 (14.0)	18 (8.9)	
GG	0 (0)	1 (0.4)	0 (0)	
A allele	196	450	388	0.107/0.064
G allele	16	36	18	
*rs8099917*				
Analyzed samples	106	241	204	
TT	90 (85.0)	215 (89.2)	192 (94.1)	0.017/0.035
TG	16 (15.0)	26 (10.8)	12 (5.9)	
GG	0 (0)	0 (0)	0 (0)	
T allele	196	456	396	0.009/0.071
G allele	16	26	12	

Variables are expressed as n or n (%).

UE, Subjects who were never exposed to HBV infection; SC, Subjects with spontaneous HBV clearance; CC, subjects with CHB or CHB-related HCC.

SNP, single-nucleotide polymorphism; HBV, hepatitis B virus, CHB, chronic hepatitis B, HCC; hepatocellular carcinoma.

Regardless of group, *rs12979860* CC was most frequently identified in more than 85% of patients in each group (85.0% in the UE, 85.9% in the SC, and 93.5% in the CC group, respectively), whereas *rs12979860* TT were not identified in any group (0% in all groups) (**Table 2**). Similarly, *rs12980275* AA and *rs8099917* TT were most frequently identified (≥85%) regardless of subject group, whereas *rs12980275* GG was identified in only one subject in the SC group and *rs8099917* GG was not identified (**Table 2**). The prevalence of CC in *rs*12979860, AA in *rs*12980275, and TT in *rs*8099917 was significantly higher in the CC group than in the UE and SC groups (all *P*<0.05) (**Table 2**). In addition, allelic frequency of three *IL28B* SNPs is also described in Table 2. All genotype frequencies of these SNPs were in Hardy-Weinberg equilibrium (*P* = 0.172, 0.382, and 0.227, respectively).

### Full Sequencing of the *IL28B* Coding Region

**Figure 1:**
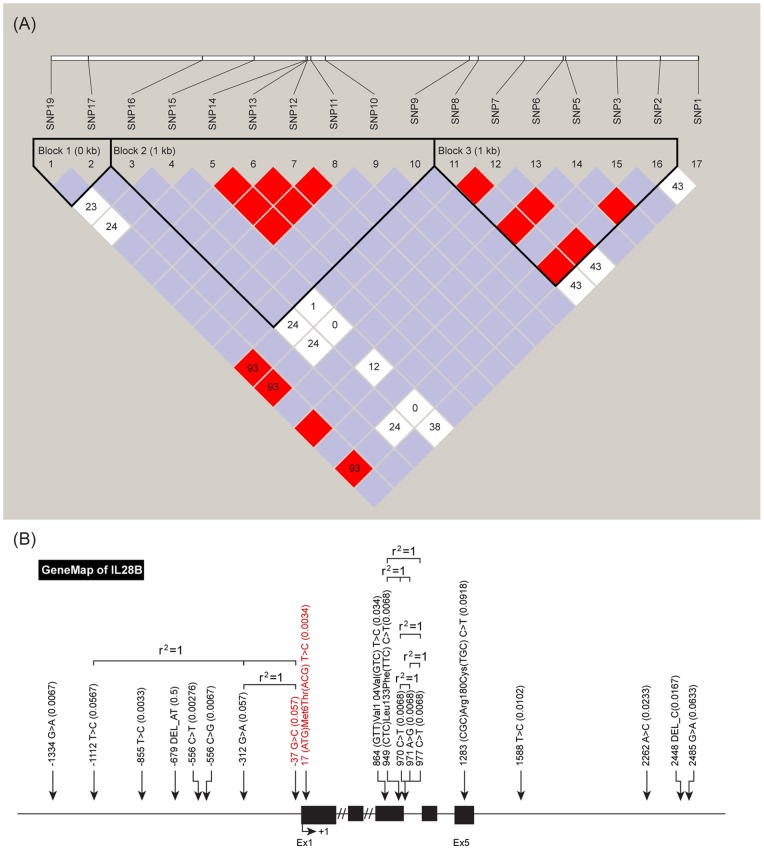
Genomic position of novel SNPs in the *IL28B* coding region (A) and linkage disequilibrium map (B). Among the 19 novel SNPs identified, the proportion of 6 SNPs (SNP 2, 5, 7, 8, 17, and 19) were significantly different among the UE, SC, and CC groups (all *P*<0.05).

Fifty samples from each group (150 samples total) were randomly selected for full sequencing of the *IL28B* coding region. The primer sequences that were used for full sequencing are described in **Table 1**. Among 19 novel SNPs that were identified in our study (**Figure 1**), the proportions of 6 SNPs (SNP 2, 5, 7, 8, 17 and 19) were significantly different among the UE, SC, and CC groups (all *P*<0.05).

### Prevalence of Newly Identified Novel SNPs in the *IL28B* Coding Region

In addition to the 150 samples that were used for full sequencing of the *IL28B* gene, 137 samples from the SC group and 109 samples from the CC group that remained after analyzing the 3 SNPs (*rs12979860*, *rs12980275*, and *rs8099917*) were additionally used for investigation of the 6 novel SNPs in the *IL28B* coding region.

Although 13.9% to 19.8% of SNaPshot analyses failed in the SC group and 66.1% to 83.5% in the CC group, SNP2 TT (84.0–86.3%), SNP5 CC (92.0–97.1%), SNP7 GG (84.0–92.7%), SNP8 GG (84.0–94.7%), SNP17 AA (94.0–98.8%), and SNP19 GG (82.0–93.0%) were most frequently identified regardless of subject group (**Table 3**). In contrast, only one case each of SNP2 CC, SNP7 AA, and SNP8 CC was identified in the CC group, whereas only one case of SNP17 GG was noted in the SC group (**Table 3**). Only the prevalence of SNP8 GG was significantly higher in the CC group (*P* = 0.026; odds ratio, 0.219, 95% CI, 0.055–0.870), where the prevalence of SNP19 GG trended higher in the CC group (*P* = 0.051). In addition, allelic frequency of six novel SNPs is also described in Table 3. All genotype frequencies of these SNPs were in Hardy-Weinberg equilibrium (*P* = 0.865, 0.715, 0.871, 0.799, 0.180, and 0.259, respectively).

**Table 3:** Prevalence of newly identified six SNPs in *IL28B* gene in UE, SC, and CC groups.

SNP	UE (n = 50)	SC (n = 162)	CC (n = 80)	UE *vs*. CC			SC *vs*. CC		
				*P* value	OR	95% CI	*P* value	OR	95% CI
**SNP2**									
TT	42 (84.0)	145 (89.5)	69 (86.3)	0.611	–	–	0.665	–	–
CT	8 (16.0)	17 (10.5)	10 (12.5)						
CC	0 (0)	0 (0)	1 (1.2)						
T allele	92	307	148	0.883			0.326		
C allele	8	17	12						
**SNP5**									
CC	46 (92.0)	158 (96.3)	66 (97.1)	0.137	–	–	0.999	–	–
CT	4 (8.0)	6 (3.7)	2 (2.9)						
TT	0 (0)	0 (0)	0 (0)						
C allele	96	322	134	0.406			0.787		
T allele	4	6	2						
**SNP7**									
GG	42 (84.0)	144 (89.4)	64 (92.7)	0.121	–	–	0.324	–	–
GA	8 (16.0)	17 (10.6)	4 (5.8)						
AA	0 (0)	0 (0)	1 (1.5)						
G allele	92	305	132	0.238			0.674		
A allele	8	17	6						
**SNP8**									
GG	42 (84.0)	143 (89.4)	72 (94.7)	0.026	0.219	0.055–0.870	0.131	–	–
GC	8 (16.0)	17 (10.6)	3 (3.9)						
CC	0 (0)	0 (0)	1 (1.4)						
G allele	92	303	147	0.098			0.330		
C allele	8	17	5						
**SNP17**									
AA	49 (98.0)	158 (94.0)	86 (98.8)	0.999	–	–	0.171	–	–
AG	1 (2.0)	9 (5.4)	1 (1.2)						
GG	0 (0)	1 (0.6)	0 (0)						
A allele	99	325	172	0.999			0.067		
G allele	1	11	1						
**SNP19**									
GG	41 (82.0)	146 (86.9)	80 (93.0)	0.051	–	–	0.203	–	–
GA	9 (18.0)	22 (13.1)	6 (7.0)						
AA	0 (0)	0 (0)	0 (0)						
G allele	91	314	16	0.055			0.153		
A allele	9	22	6						

Variables are expressed as n (%).

UE, Subjects who were never exposed to HBV infection; SC, Subjects with spontaneous HBV clearance;

CC, subjects with CHB or CHB-related HCC.

SNP, single-nucleotide polymorphism; HBV, hepatitis B virus, CHB, chronic hepatitis B, HCC; hepatocellular carcinoma; CI, confidence interval.

### Haplotypic Combinations

Haplotypic combination using *rs12979860*, *rs12980275*, and *rs8099917* did not show statistically better odds ratio (all *P*>0.05). However, when we performed haplotypic combination analysis using *rs12979860*, *rs12980275*, *rs8099917* and newly identified 19 SNPs, four haplotypic combinations were significantly different between SC and CC groups (Table 4).

**Table 4:** Haplotypic analysis.

Combination of SNPs	Haplotypes	*P* value (SC vs. CC group)
*rs12980275*-SNP19	T-G	0.012
SNP8-SNP7	G-G	0.015
*rs12979860*–*rs8099917*	G-T	0.006
SNP8-SNP7-SNP6-SNP5-SNP3-SNP2-SNP1-rs12979860-*rs8099917*	G-G-C-C-T-T-G-G-T	0.029

## Discussion

*IL28B* (IFN λ3) triggers a cascade through the JAK-STAT pathway that upregulates the IFN-stimulated genes (ISGs) [13], [14]. The effects of *IL28B* are similar to those of type I IFNs such as IFN-α and -β; however, *IL28B* binds to a distinct receptor that may modulate a different set of ISGs [15]. Recently, this cytokine was identified as a key factor of the immune response to HCV. Indeed, there has been increasing evidence showing that an *IL28B* haplotype strongly determines both the outcome of natural and IFN-α-treated HCV infection [1]–[5].

Because IFN-α and ISGs are thought to be important in the immune response to HBV, and PEG-IFN-α is used to treat chronic HBV infection similar to HCV infection, we can assume that *IL28B* may also be important in treatment and recovery from HBV infection. Currently, two reports investigated the clinical role of the *IL28B* polymorphism in HBV infection are currently available [7], [12]. One study investigated whether the *IL28B* polymorphism is associated with responses to PEG-IFN in patients with hepatitis B e antigen positive CHB and proposed that polymorphisms near *IL28B* are independently associated with serological responses to PEG-IFN in CHB [7]. The other study investigated whether the polymorphism marking the haplotype (*rs12979860*) also affects other INF-α responsive chronic viral illness, namely HBV, and concluded that the *rs12979860* CC genotype was not associated with spontaneous HBV recovery [12]. Therefore, in contrast to HCV infection, the *IL28B* polymorphism seems to act via different mechanism in terms of treatment outcomes using INF-α and spontaneous clearance in HBV infection.

In our study, to determine the potential influence of the *IL28B* polymorphism on HBV infection outcome in a natural history setting, we genotyped this polymorphism in HBV cohorts comprising subjects who spontaneously cleared HBV or had persistent infection and those who were never exposed to HBV infection. In contrast to a previous study [12], our study demonstrated that *IL28B* polymorphisms (*rs12979860*, *rs12980275*, and *rs8099917*) are significantly associated with the outcomes of HBV infection (**Table 2**). However, the prevalence of the major alleles *rs12979860* CC, *rs12980275* AA, and *rs8099917* TT (>85%), which are favorable predictors in terms of spontaneous clearance of HCV infection, were unexpectedly higher in subjects who did not clear HBV than in those who experienced spontaneous clearance of HBV or those without exposure to HBV infection. This high prevalence of the major *IL28B* polymorphism alleles has been reported in several previous Korean studies [16] and is consistently higher than the reported prevalences from Western studies [9]. Based on this finding, higher SVR rates in Korean patients with CHC have recently been reported [16]. However, because IFN-based treatment outcomes have not been investigated in Korean patients with CHB showing a high prevalence of major *IL28B* polymorphism alleles, further studies are needed to investigate whether the IFN-based treatment outcomes for CHB are similar to those of Western data adjusting for the influence of HBV genotype (nearly 100% of genotype C in Korea).

Considering that prior studies have shown that *IL28B* polymorphism is associated with spontaneous and treatment-induced clearance of HCV and has one of the strongest known genetic associations with any chronic viral infection to date [1]–[5], we can assume that *IL28B* polymorphisms have a distinct effect on the immune response to HCV in spite of the same signal from both type I IFNs and *IL28B* through the JAK-STAT pathway. Because ISGs were shown to be a major mechanism of non-cytolytic inhibition of HBV replication in a transgenic mouse model [17] and IFN-λ2 (IL28A) inhibits HBV replication through upregulated ISGs in HCC cell lines [18], it is difficult to explain the negative correlation between major alleles of *IL28B* polymorphism and HBV spontaneous clearance given that *IL28B* stimulates ISGs, which play an important role in the immune response to HBV infection. However, differences between HBV and HCV infection in terms of their replication strategies, pathways to viral persistence and clearance, and host responses, including the production of type I IFNs, are well known [19]. In addition, several unique characteristics of *IL28B* may explain this unexpected finding. In addition to inducing ISG expression, *IL28B* may activate alternate antiviral pathways, such as the adaptive immune response, which may be more important in HCV [20]. *IL28B* may also lead to a different antiviral state than type I IFNs, in terms of the upregulation of different ISGs and different speeds of phosphorylation of STAT1 and STAT2 [15]. It is also possible that the *IL28B* pathway may respond differently to HCV and HBV (more dominant in HCV), which can lead to a different effect on HCV and HBV infection. Some previous studies support this hypothesis by proposing lower antiviral activities of IFN- λ2 (IL28A) and IFN-λ1 (IL29) [16], [18], although extrapolation of these findings to *IL28B* should be determined. Finally, it is also plausible that the *IL28B* and IFN-α pathways are synergistic because of common downstream signaling pathways and that this synergism results in different immune responses to HCV and HBV. However, all of these assumptions, including a lack of influence of the major *IL28B* polymorphism alleles on HBV clearance [10] or the negative influence observed in our study, should be validated in future work.

Interestingly, we found one novel SNP in the *IL28B* gene (SNP8) that was located immediately ahead of the *IL28B* exon 1 (**Figure 1**). Its major allele (GG) prevalence was significantly higher in subjects with persistent HBV infection (**Table 3**), although we failed to obtain SNP data in a number of cases using the SNaPshot assay, possibly because of high homology in the nucleotide arrangement on chromosome 19. Thus, we could assume that the genomic site of the SNP8 polymorphism might serve as a binding area for certain transcriptional factors or polymerases and may therefore control gene expression levels by regulating binding capacity. However, functional studies such as reporter assays are needed to validate this assumption. In addition, four haplotypic combinations were significantly different between SC and CC groups. However, further studies are required to reveal the clinical relevance of these haplotypic combinations.

Our study has several strengths. First, this is the first Korean study to focus on the influence of the *IL28B* polymorphism on the natural clearance of HBV infection and to investigate *IL28B* polymorphisms in a healthy Korean population. Thus, our results can help establish the worldwide database of *IL28B* polymorphisms not only for subjects with persistent HBV infection, but also for apparently healthy populations. Second, although the *IL28B* polymorphism has been known to have a significant correlation with IFN-based treatment of HCV and spontaneous clearance of HCV infection, how this polymorphism influences gene expression levels relatively far from the actual *IL28B* coding region has been unclear. Thus, in our study, we attempted to sequence the full *IL28B* coding region using highly qualified PCR techniques with multiple primers to investigate whether there is any novel polymorphism in the actual *IL28B* coding region that might be related to the clearance of HBV infection. However, because only native Korean subjects were recruited for this study, our results should be interpreted with cautions and external validation in other ethnic groups should be followed. Furthermore, anti-HBs information was not available in our study, hence the correlation between *IL28B* SNP and anti-HBs could not be analyzed.

In conclusion, the SNP upstream of *IL28B* that has the strongest genetic association with HCV recovery to date inversely influences on HBV recovery. Thus, the effects of this SNP cannot be generalized to chronic viral infections in which IFN-α treatment is important. Additional studies are needed to understand the mechanisms underlying the effects of this SNP in HBV infection.

## References

[1] Ge D, Fellay J, Thompson AJ, Simon JS, Shianna KV, et al (2009) Genetic variation in IL28B predicts hepatitis C treatment-induced viral clearance. Nature 461: 399–401. 19684573 10.1038/nature08309

[2] Suppiah V, Moldovan M, Ahlenstiel G, Berg T, Weltman M, et al (2009) IL28B is associated with response to chronic hepatitis C interferon-alpha and ribavirin therapy. Nat Genet 41: 1100–1104. 19749758 10.1038/ng.447

[3] Tanaka Y, Nishida N, Sugiyama M, Kurosaki M, Matsuura K, et al (2009) Genome-wide association of IL28B with response to pegylated interferon-alpha and ribavirin therapy for chronic hepatitis C. Nat Genet. 41: 1105–1109. 10.1038/ng.449 19749757

[4] Rauch A, Kutalik Z, Descombes P, Cai T, Di Iulio J, et al.. (2010) Genetic variation in IL28B is associated with chronic hepatitis C and treatment failure: a genome-wide association study. Gastroenterology 138: 1338–1345, 1345.e1–7. 10.1053/j.gastro.2009.12.056 20060832

[5] Ank N, West H, Bartholdy C, Eriksson K, Thomsen AR, et al (2006) Lambda interferon (IFN-lambda), a type III IFN, is induced by viruses and IFNs and displays potent antiviral activity against select virus infections in vivo. J Virol 80: 4501–4509. 16611910 10.1128/JVI.80.9.4501-4509.2006 PMC1472004

[6] Ank N, West H, Paludan SR (2006) IFN-lambda: novel antiviral cytokines. J Interferon Cytokine Res 26: 373–379. 16734557 10.1089/jir.2006.26.373

[7] Sonneveld MJ, Wong VW, Woltman AM, Wong GL, Cakaloglu Y, et al (2012) Polymorphisms Near IL28B and Serologic Response to Peginterferon in HBeAg-Positive Patients With Chronic Hepatitis B. Gastroenterology. 142: 513–520.e1. 10.1053/j.gastro.2011.11.025 22108195

[8] Lampertico P, Viganò M, Cheroni C, Facchetti F, Invernizzi F, et al.. (2012) IL28B polymorphisms predict interferon-related hepatitis B surface antigen seroclearance in genotype D hepatitis B e antigen-negative patients with chronic hepatitis B. Hepatology Apr 2. doi: 10.1002/hep.25749. [Epub ahead of print] 10.1002/hep.25749 22473858

[9] Thomas DL, Thio CL, Martin MP, Qi Y, Ge D, et al (2009) Genetic variation in IL28B and spontaneous clearance of hepatitis C virus. Nature 461: 798–801. 19759533 10.1038/nature08463 PMC3172006

[10] Montes-Cano MA, García-Lozano JR, Abad-Molina C, Romero-Gómez M, Barroso N, et al (2010) Interleukin-28B genetic variants and hepatitis virus infection by different viral genotypes. Hepatology 52: 33–37. 20578254 10.1002/hep.23624

[11] Tillmann HL, Thompson AJ, Patel K, Wiese M, Tenckhoff H, et al.. (2010) A polymorphism near IL28B is associated with spontaneous clearance of acute hepatitis C virus and jaundice. Gastroenterology 139: 1586–1592, 1592.e1. 10.1053/j.gastro.2010.07.005 20637200

[12] Martin MP, Qi Y, Goedert JJ, Hussain SK, Kirk GD, et al (2010) IL28B polymorphism does not determine outcomes of hepatitis B virus or HIV infection. J Infect Dis 202: 1749–1753. 20977343 10.1086/657146 PMC2974014

[13] Honda M, Sakai A, Yamashita T, Nakamoto Y, Mizukoshi E, et al (2010) Hepatic ISG expression is associated with genetic variation in interleukin 28B and the outcome of IFN therapy for chronic hepatitis C. Gastroenterology. 139: 499–509. 10.1053/j.gastro.2010.04.049 20434452

[14] Urban TJ, Thompson AJ, Bradrick SS, Fellay J, Schuppan D, et al (2010) IL28B genotype is associated with differential expression of intrahepatic interferon-stimulated genes in patients with chronic hepatitis C. Hepatology. 52: 1888–1896. 10.1002/hep.23912 PMC3653303 20931559

[15] Marcello T, Grakoui A, Barba-Spaeth G, Machlin ES, Kotenko SV, et al (2006) Interferons alpha and lambda inhibit hepatitis C virus replication with distinct signal transduction and gene regulation kinetics. Gastroenterology 131: 1887–1898. 17087946 10.1053/j.gastro.2006.09.052

[16] Hong SH, Cho O, Kim K, Shin HJ, Kotenko SV, et al (2007) Effect of interferon-lambda on replication of hepatitis B virus in human hepatoma cells. Virus Res 126: 245–249. 17451832 10.1016/j.virusres.2007.03.006

[17] Guidotti LG, Morris A, Mendez H, Koch R, Silverman RH, et al (2002) Interferon-regulated pathways that control hepatitis B virus replication in transgenic mice. J Virol 76: 2617–2621. 11861827 10.1128/JVI.76.6.2617-2621.2002 PMC135990

[18] Robek MD, Boyd BS, Chisari FV (2005) Lambda interferon inhibits hepatitis B and C virus replication. J Virol 79: 3851–3854. 15731279 10.1128/JVI.79.6.3851-3854.2005 PMC1075734

[19] Wieland SF, Chisari FV (2005) Stealth and cunning: hepatitis B and hepatitis C viruses. J Virol 79: 9369–9380. 16014900 10.1128/JVI.79.15.9369-9380.2005 PMC1181548

[20] Morrow MP, Pankhong P, Laddy DJ, Schoenly KA, Yan J, et al (2009) Comparative ability of IL-12 and IL-28B to regulate Treg populations and enhance adaptive cellular immunity. Blood 113: 5868–5877. 19304955 10.1182/blood-2008-11-190520 PMC2700323

